# M6A RNA methylation modification and tumor immune microenvironment in lung adenocarcinoma

**DOI:** 10.52601/bpr.2023.220020

**Published:** 2023-06-30

**Authors:** Shujuan Li, Qianzhong Li, Luqiang Zhang, Yechen Qi, Hui Bai

**Affiliations:** 1 Laboratory of Theoretical Biophysics, School of Physical Science and Technology, Inner Mongolia University, Hohhot 010021, China; 2 The State Key Laboratory of Reproductive Regulation and Breeding of Grassland Livestock, Inner Mongolia University, Hohhot 010070, China

**Keywords:** Lung adenocarcinoma, M6A RNA methylation regulators, Consensus clustering, Immune cell infiltrates, Tumor immune microenvironment

## Abstract

Lung adenocarcinoma is one of the deadliest tumors. Studies have shown that N6-methyladenosine RNA methylation regulators, as a dynamic chemical modification, affect the occurrence and development of lung adenocarcinoma. To investigate the relationship between mutations and expression levels of m6A regulators in lung adenocarcinoma, we investigated the mutations and expression levels of 38 m6A regulators. We found that mutations in m6A regulatory factors did not affect the changes in expression levels, and 19 differentially expressed genes were identified. All tumor samples were classified into two subtypes based on the expression levels of 19 differentially expressed m6A-regulated genes. Survival analysis showed significant differences in survival between the two subtypes. To explore the relationship between immune cell infiltration and survival in both subtypes, we calculated the infiltration of 23 immune cells in both subtypes, and we found that the subtype with high immune cell infiltration had better survival. We found that subtypes with low tumor purity and high stromal and immune scores had better survival. The m6A-related immune genes were identified by taking the intersection of differentially expressed genes and immune genes in the two isoforms and calculating the Pearson correlation coefficients between the intersecting immune genes and the differentially expressed m6A-regulated genes. Finally, a prognostic model associated with m6A and associated with immunity was developed using prognostic genes screened from m6A-associated immune genes. The predictive power of the model was evaluated and our model was able to achieve good prediction.

## INTRODUCTION

Lung cancer is one of the most common malignant tumors with high mortality in the world, and it is characterized by late diagnosis (Hirsch *et al.* 2017). Lung cancer is the second most common cancer and the leading cause of cancer death in 2022 (Siegel *et al.* 2022). The most common type of lung cancer is lung adenocarcinoma (LUAD), which accounts for about 40% of all lung cancer cases and it is one of the most aggressive and rapidly fatal tumor types, with an overall survival of less than five years (Denisenko *et al.* 2018). Despite vast improvements in diagnostic and therapeutic techniques, the prognosis for patients with LUAD remains poor (Dolly *et al.* 2017). Therefore, it is crucial to identify therapeutic targets and effective prognostic factors to improve the prognosis of patients with LUAD.

The m6A is a post-transcriptional chemical modification that occurs on RNA, and methylation occurs on the sixth nitrogen of the purine ring (Khan and Malla 2021). As one of the most common chemical modifications in mRNA in eukaryotes, m6A maintains the stability of eukaryotic mRNA and affects mRNA splicing, translation, nuclear export and degradation (Barbieri and Kouzarides 2020). Among them, the m6A modification on mRNA is the most abundant, and the research on m6A modification on mRNA is also the most. Studies have shown that m6A modification plays a pivotal role in the occurrence and development of lung adenocarcinoma, and has become one of the hotspots in tumor biology research. The m6A methylation modification on mRNA had been used as a biomarker for the timely screening and treatment of lung adenocarcinoma (Khan and Malla 2021). Therefore, studying the biological function of m6A modification in lung adenocarcinoma is of great significance for elucidating the pathogenesis of lung adenocarcinoma and providing a theoretical basis for the clinical treatment of lung adenocarcinoma.

The modification of RNA m6A is involved in many important biological processes, including gene expression, immune regulation, and cancer (Fang *et al.* 2020). The tumor immune microenvironment (TME) constitutes the surrounding area during tumor development, is the product of mutual interference between different cell types, and plays a crucial role in tumor progression, metastasis and treatment (Baghban *et al.* 2020; Hanahan and Coussens 2012; Hu and Polyak 2008; Zhang *et al.* 2020b). The TME is important for the formation, progression and treatment of tumors containing tumor cells, immune cells and stromal cells (Do *et al.* 2020). Stromal cells in the TME are attractive therapeutic targets to reduce the risk of drug resistance and tumor recurrence (Quail and Joyce 2013). Studies have shown that immune cells in the tumor microenvironment are closely related to tumor prognosis and malignancy, and TME has become a research hotspot in the field of tumors (Hanahan and Coussens 2012). Research shows that the tumor microenvironment of the lung can be targeted for therapy, and related drugs are being studied in clinical trials (Altorki *et al.* 2019). Therefore, it is extremely urgent to investigate the m6A methylation modification and the immune microenvironment in lung adenocarcinoma to study the occurrence and development of lung adenocarcinoma. Although there are many studies on m6A modification in lung adenocarcinoma, almost all are based on the experimental level.

In this study, we assessed the expression and mutation of 38 m6A regulators in the TCGA database. Lung adenocarcinoma patients were divided into two subtypes based on differentially expressed genes. We analyzed and compared immune cell infiltration and tumor purity between different subtypes. We screened out the prognostic immune genes associated with m6A to establish a prognostic risk score model. These results have important implications for the diagnosis and treatment of LUAD.

## RESULTS

### Mutation and expression of m6A regulators in LUAD

**Figure 1:**
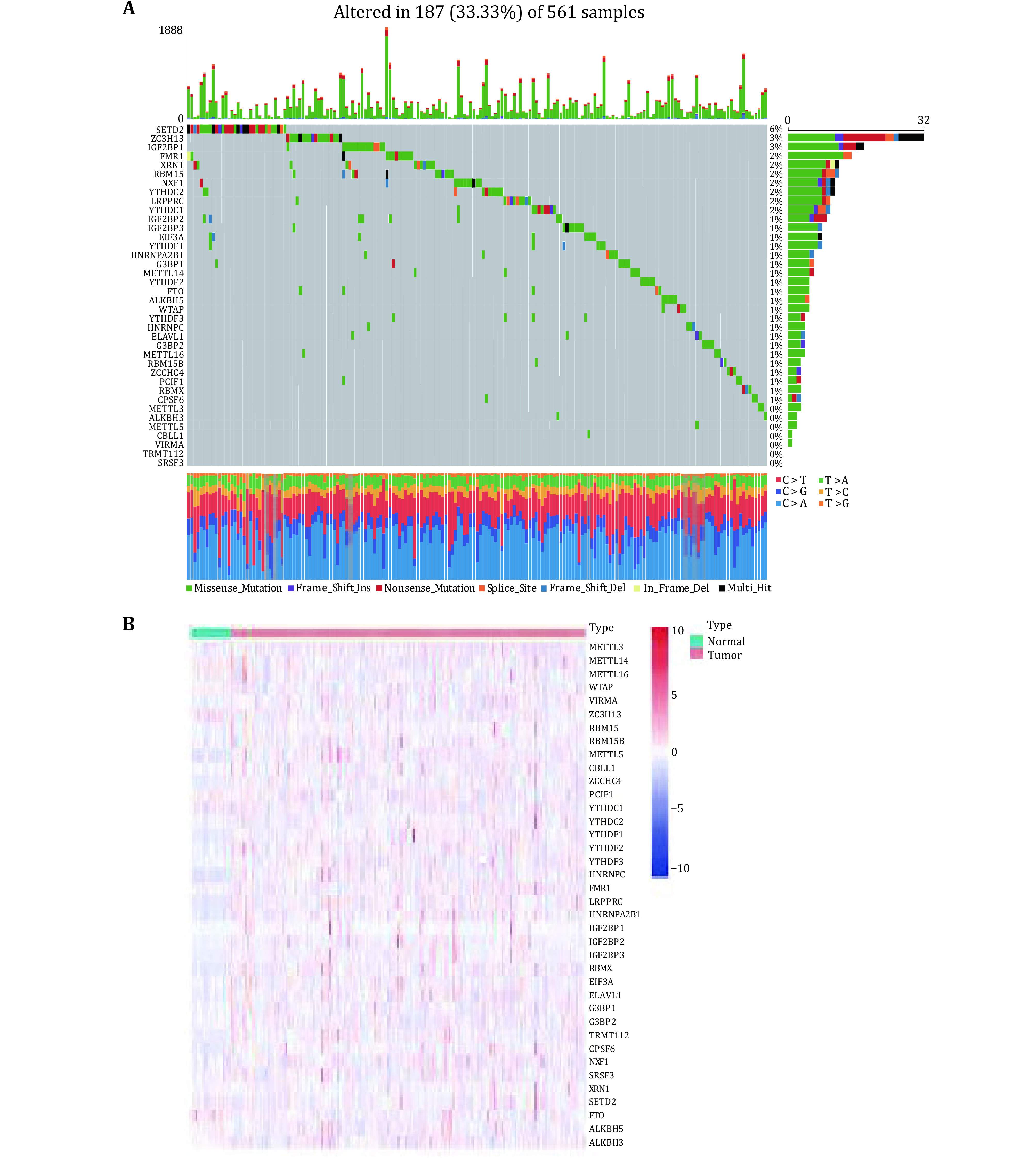
Expression levels of m6A RNA methylation regulators and mutation frequency. **A** Mutation frequency levels of 38 m6A regulators in LUAD. **B** The heat map shows the expression levels of 38 m6A regulators in LUAD and normal samples

We investigated the incidence of somatic mutations in 38 m6A regulators in lung adenocarcinoma. As shown in Fig. 1A, 187 of the 561 lung adenocarcinoma samples had mutations (mutation frequency 33.33%). In the mutated LUAD samples, a total of 31 genes were mutated, with the m6A regulator *SETD2* showing the highest mutation frequency of only 6%. The mutation frequency of 21 m6A-regulated genes was about 1%, and about seven m6A-regulated genes showed no mutation (Fig. 1A). To determine whether genetic mutations affect the expression patterns of m6A regulators in LUAD samples, the mRNA expression levels of 38 regulators between LUAD and normal lung adenocarcinoma samples were visualized as a heatmap (Fig. 1B). The overall distribution showed that the expression levels of 38 m6A regulators were inconsistent in tumor and normal samples. Some m6A regulators were lowly expressed in normal samples, some were highly expressed in tumor samples, and some genes were not differentially expressed between normal and tumor samples.

### Lung adenocarcinoma subtypes based on differentially expressed m6A gene

**Figure 2:**
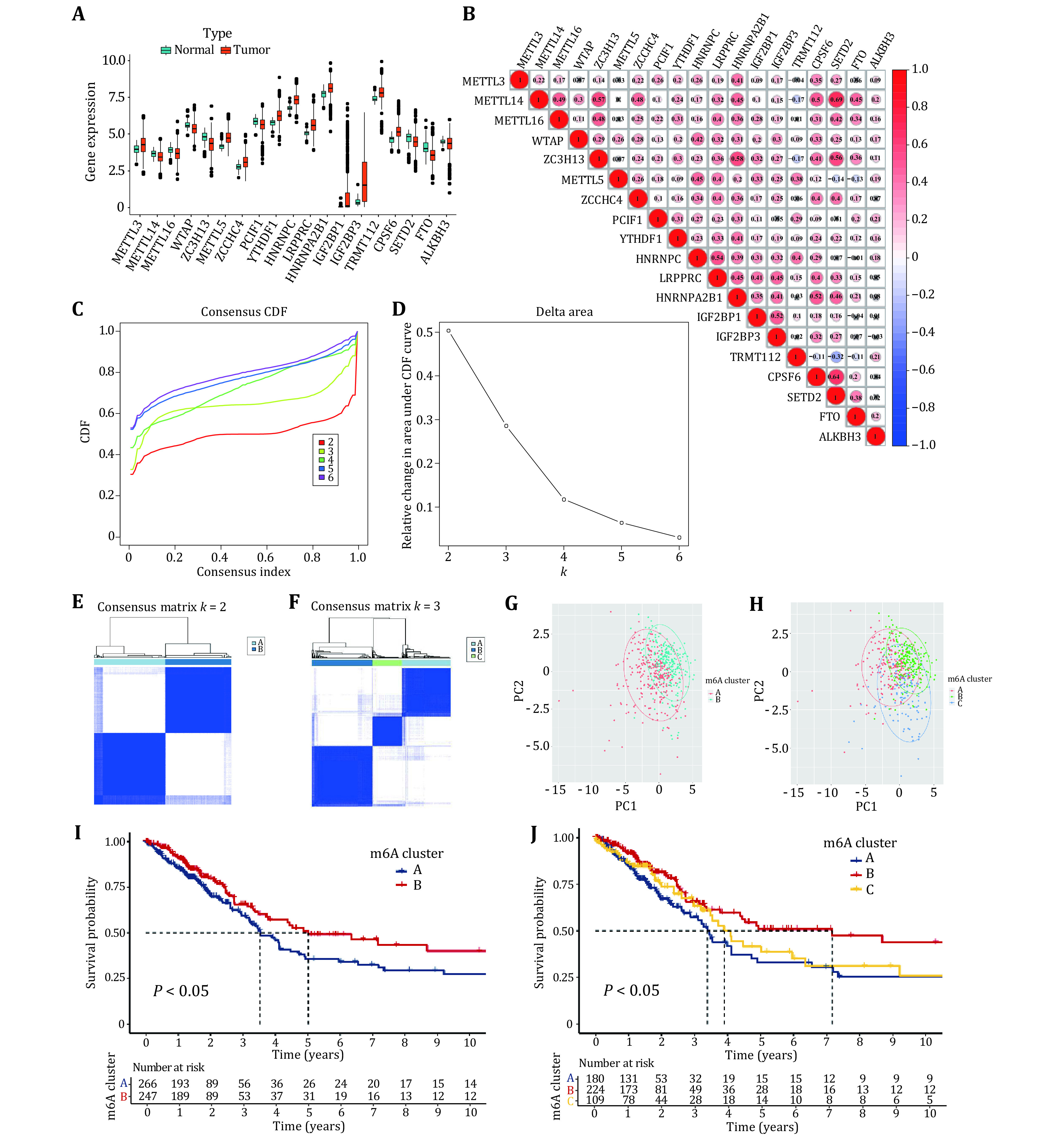
Different analysis of m6A RNA methylation regulators and consensus clustering analysis. **A** Expression distributions of m6A regulators in tumor and normal samples. **B** Co-expression patterns of 19 differentially expressed m6A regulators. Negative correlations are marked with blue, and positive correlations are marked with red. **C** Consensus clustering CDF for *k* = 2, 3, 4, 5 and 6. **D** CDF curve for consensus clustering. **E** Consensus clustering of LUAD patients for *k* = 2. **F** Consensus clustering CDF for *k* = 3. **G** Principal component analysis for patients in Clusters A and B. **H** Principal component analysis for patients in Clusters A, B and C. **I** Survival analyses for patients in Clusters A and B. **J** Survival analyses for patients in Clusters A, B and C

The differentially expressed genes were found according to the m6A regulator expression values in normal and tumor samples. The 19 differentially expressed m6A-regulated genes with FDR < 0.05 and $ { {\log}}_{ {2}}\left( {folding\;\; change}\right) < - {0.25} $ or $ \geqslant  {0.25} $, including eight down-regulated m6A-regulated genes (FDR < 0.05 and $ { {\log}}_{ {2}}\left( {folding\;\; change}\right)  <  - {0.25} $) and 11 up-regulated m6A-regulated genes (FDR < 0.05 and $ { {\log}}_{ {2}}\left( {folding\;\;change}\right)  \geqslant   {0.25} $) were obtained. The remaining m6A-regulated genes were not differentially expressed in normal and tumor samples (supplementary Table S1). It can be seen from Fig. 2A that the differentially expressed m6A-regulated genes *METTL3*, *METTL5*, *ZCCHC4*, *YTHDF1*, *HNRNPC*, *LRPPRC*, *HNRNPA2B1*, *IGF2BP1*, *IGF2BP3*, *TRMT112* and *CPSF6* are highly expressed in tumor samples; genes *METTL14*, *METTL16*, *WTAP*, *ZC3H13*, *PCIF1*, *SETD2*, *FTO* and *ALKBH3* were lowly expressed in tumor samples. The detailed information including average expression levels, differential fold, and corrected *P* values for the 38 m6A-regulated genes were shown in the supplementary Table S1. Subsequently, we explored the expression relationship of 19 differentially expressed m6A regulators in lung adenocarcinoma. Notably, significant correlations were observed between the expression of methyltransferases, demethylases and binding proteins (Fig. 2B). For example, *SETD2* was positively correlated with *METTL14*, and the correlation reaches 0.69. *ZC3H13* was positively related to *METTL14*, *HNRNPA2B1* and *SETD2*, and the correlation coefficients were all greater than 0.5. *TRMT112* was negatively correlated with *METTL14, ZC3H13, CPSF6, SETD2* and *FTO*, and *METTL5* was negatively correlated with *SETD2* and *FTO*. Then, all tumor samples were classified into two classes based on 19 differentially expressed m6A regulators by consensus clustering. Figs. 2C and 2D show that the relative change in area under the cumulative distribution function (CDF) curve is small when *k* = 3, indicating that *k* = 3 is a reasonable type. However, Figs. 2E and 2F show that the sample size is too small for one of the three subtypes, so it is reasonable to consider splitting it into two subtypes. As in Figs. 2G and 2H cannot well justify the division into two subtypes or three subtypes. To better illustrate the rationality of the typing, we used the Kaplan-Meier survival curve. Figure 2I shows that the survival rate of Subtype B is better than that of Subtype A. Figure 2J shows that when tumor samples were divided into three subtypes, the survival of Subtype C was intermediate between Subtypes A and B. Therefore, the division into two subtypes is reasonable.

### Immune cell infiltration and tumor purity among different subtypes of lung adenocarcinoma

**Figure 3:**
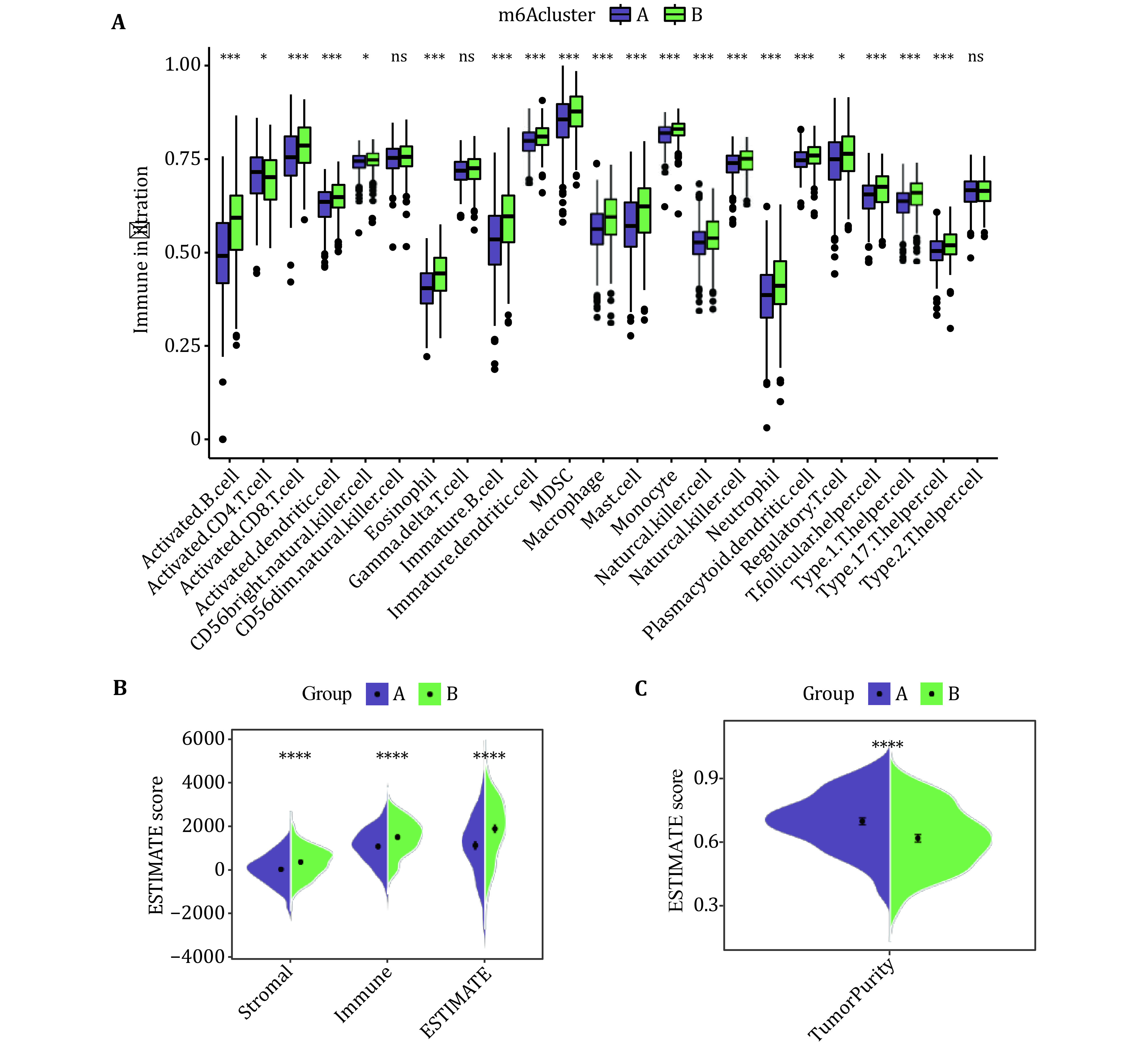
Tumor immune microenvironment between Subtype **A** and Subtype B patients with LUAD. A Infiltration of 23 immune cells in Subtypes A and B. **B** The estimated score of immune cells and stromal cells between Subtype A and Subtype B. **C** The estimated score of tumor purity between Subtype A and Subtype B

To explore the differences in the tumor immune microenvironment between the two different subtypes, the infiltration levels of 23 immune cell types were assessed by using ssGSEA (Fig. 3A). We calculated the infiltration levels of stromal and immune scores between the two subtypes using the ESTIMAIE algorithm. Compared with the Subtype A, the Subtype B had higher immune and stromal scores (Fig. 3B). In addition, it is speculated that the tumor purity of Subtype A is higher than that of Subtype B. The tumor purity results calculated by the "affymetrix" platform were shown to be consistent with the predicted results (Fig. 3C).

### Screening of m6A-related immune genes

**Figure 4:**
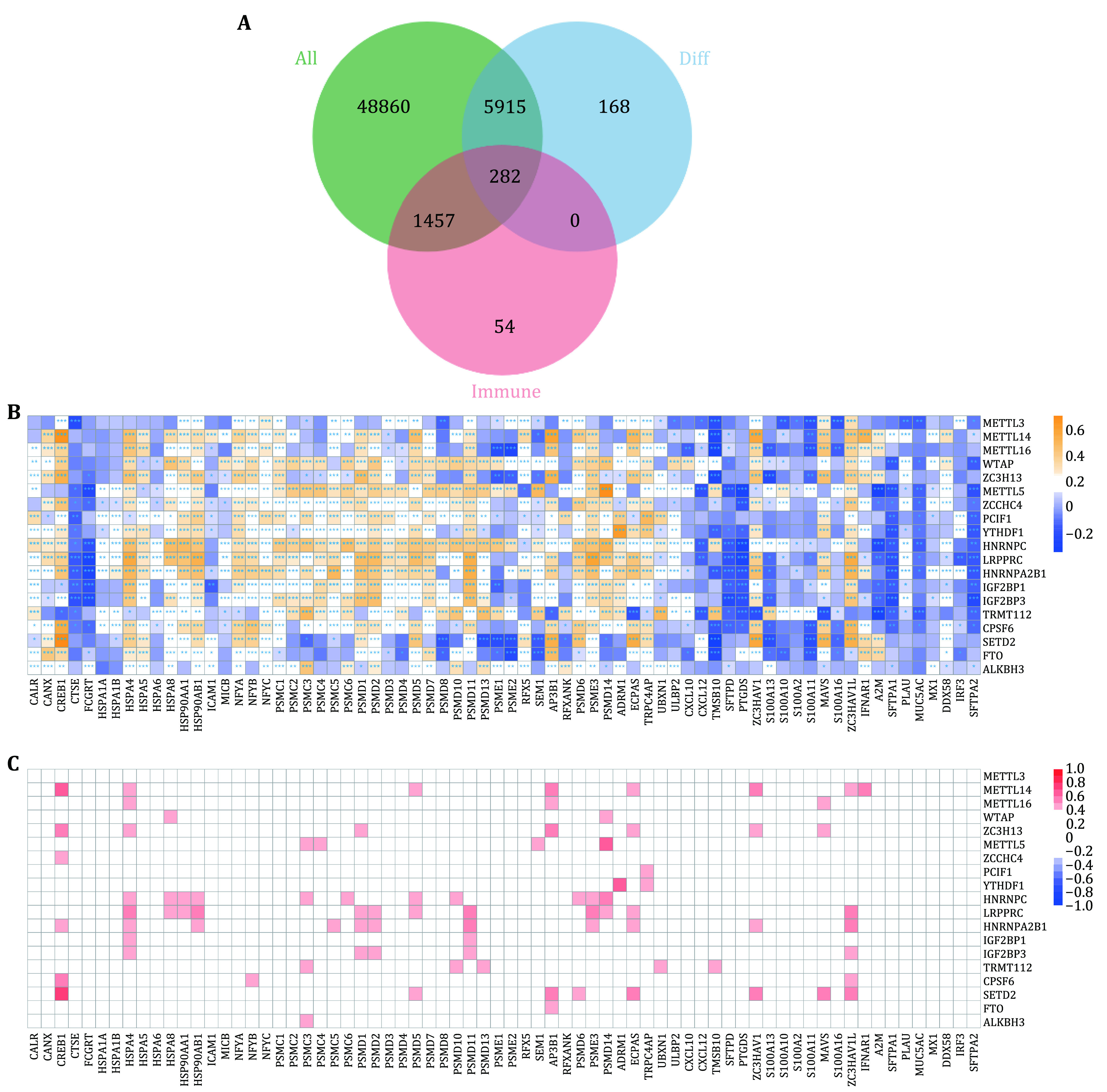
Association analysis of immune genes and m6A regulators. **A** Selection of differentially expressed immune genes. **B** Pearson correlation between 19 differentially expressed m6A regulators and 282 immune genes. **C** Visualization of genes whose coefficients were greater than 0.1 and *P*.adj < 0.05, which were called m6A-related immune genes

Studies have shown that m6A RNA methylation regulators are significantly related to immune infiltration and prognosis of lung adenocarcinoma. Therefore, we established an immune-related prognostic model based on m6A-related genes for the prognosis of lung adenocarcinoma. There were 56,514 genes in each tumor sample. After filtering out genes that were not expressed in all samples, 43,863 genes were retained. By intersecting the differential expressed genes and immune genes, 282 genes were extracted (Fig. 4A). Then, the correlation between 282 immune genes and 19 differentially expressed m6A regulatory genes were calculated and shown in Fig. 4B, Fig. 4C, and the supplementary Figs. S1 and S2.

### Establishment of m6A-related immune prognosis model

**Figure 5:**
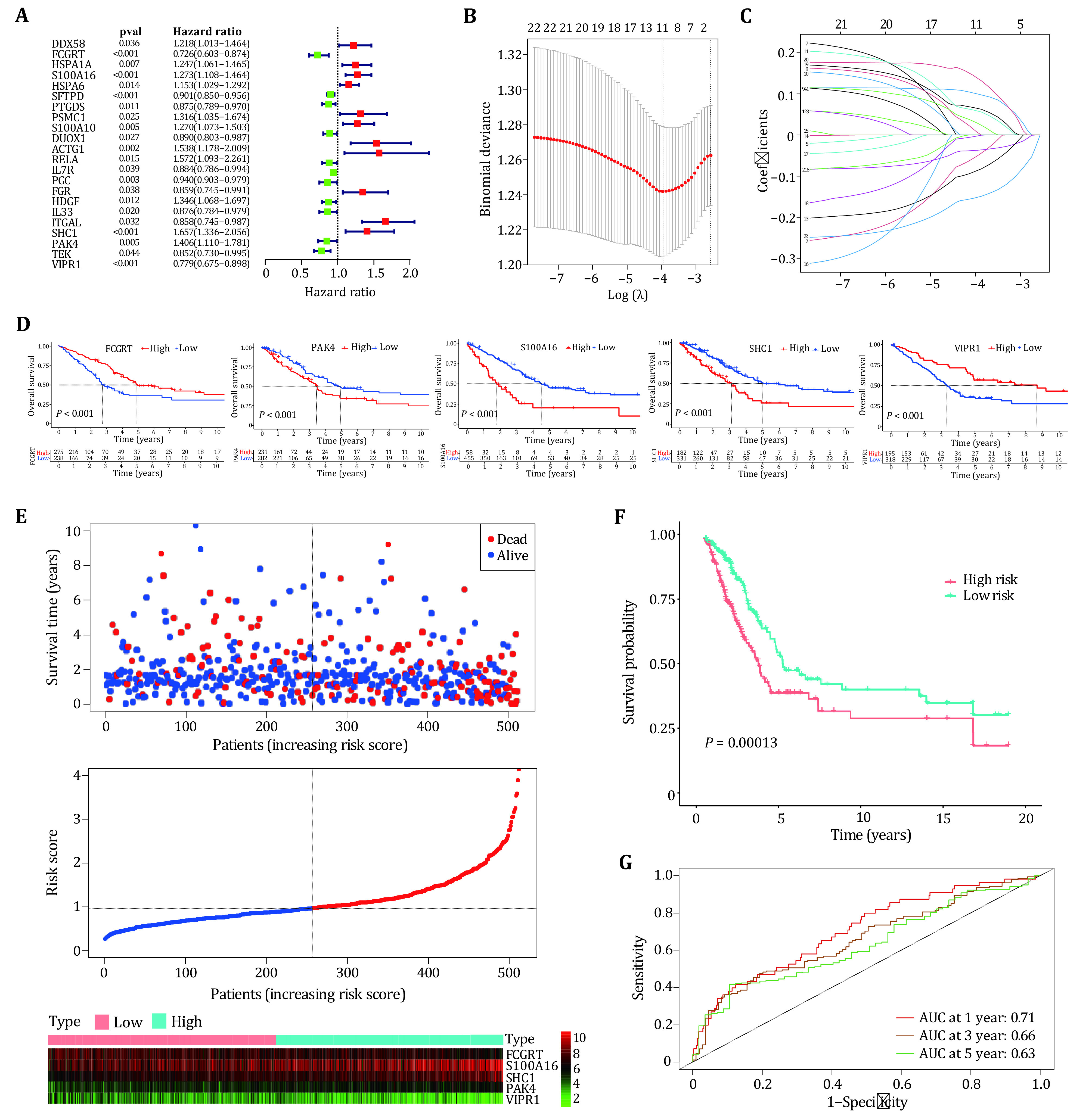
Construction of prognostic models based on m6A-related immune genes. **A** Univariate Cox regression analysis of 22 immune genes associated with m6A. **B**,**C** Least Absolute Shrinkage and Selection Operator (LASSO) algorithm narrows the range of immune prognostic genes. **D** The performance of five immune prognostic genes in patient prognosis. Wherein "high" represents high expression and "low" represents low expression. **E** Survival status, risk score, and expression distribution of five immune prognostic genes. High-risk and low-risk patients are separated by dashed lines. **F** Kaplan-Meier analysis of overall survival in the high-risk and low-risk groups. **G** ROC curves of five-gene prognostic models

To investigate the prognostic value of m6A-related genes in lung adenocarcinoma, an immune-related prognostic model was developed by using the m6A-related immune genes. We performed univariate cox regression analysis based on the expression levels of 282 genes and found 22 genes whose *P*-values were less than 0.05, including 11 risk genes (HR ≥ 1) and 11 protective genes (HR < 1) (Fig. 5A). Next, by using the lasso algorithm, the candidate genes were narrowed down to 11 for multivariate cox regression analysis, and the optimal lambda value was 0.02084 (Figs. 5B and 5C). Finally, five m6A-related prognostic immune genes were picked out and submitted to construct a prognostic model by the multivariate Cox regression analysis.

\begin{equation*}\begin{split} {risk \;score} =&-{0.1931} \times {FCGRT} + {0.121878} \times {S100A16}\\& + {0.365108} \times {SHC1} + {0.186964} \times {PAK4} \\&- {0.13349} \times {VIPR1} \end{split}\end{equation*}

Studies showed that immune genes interacting with m6A regulators can promote the occurrence and development of cancer (Li *et al.* 2021; Shulman and Stern-Ginossar 2020). Survival analysis was performed for five genes included in the prognostic model, Kaplan-Meier survival curves showed the prognosis of five genes in patients, where “high” indicates high expression and “low” indicates low expression (Fig. 5D). The survival status, risk score and expression distribution of five genes in training samples were shown in Fig. 5E. The median risk score was used to divide patients into high-risk group and low-risk group, patients with scores greater than the median were considered high-risk groups with poor prognosis, and patients with scores below median were considered low-risk groups with good prognosis (Fig. 5F). To test the applicability of the five-gene model, we drawn the receiver operating characteristic curve (ROC) in the training cohort. Figure 5G shows that the AUC value for one year is 0.71, the AUC value for three years is 0.66, and the AUC value for five years is 0.63. A prognostic model based on five m6A-related immune genes can predict survival in lung adenocarcinoma patients with moderate accuracy.

### Independent verification of models

**Figure 6:**
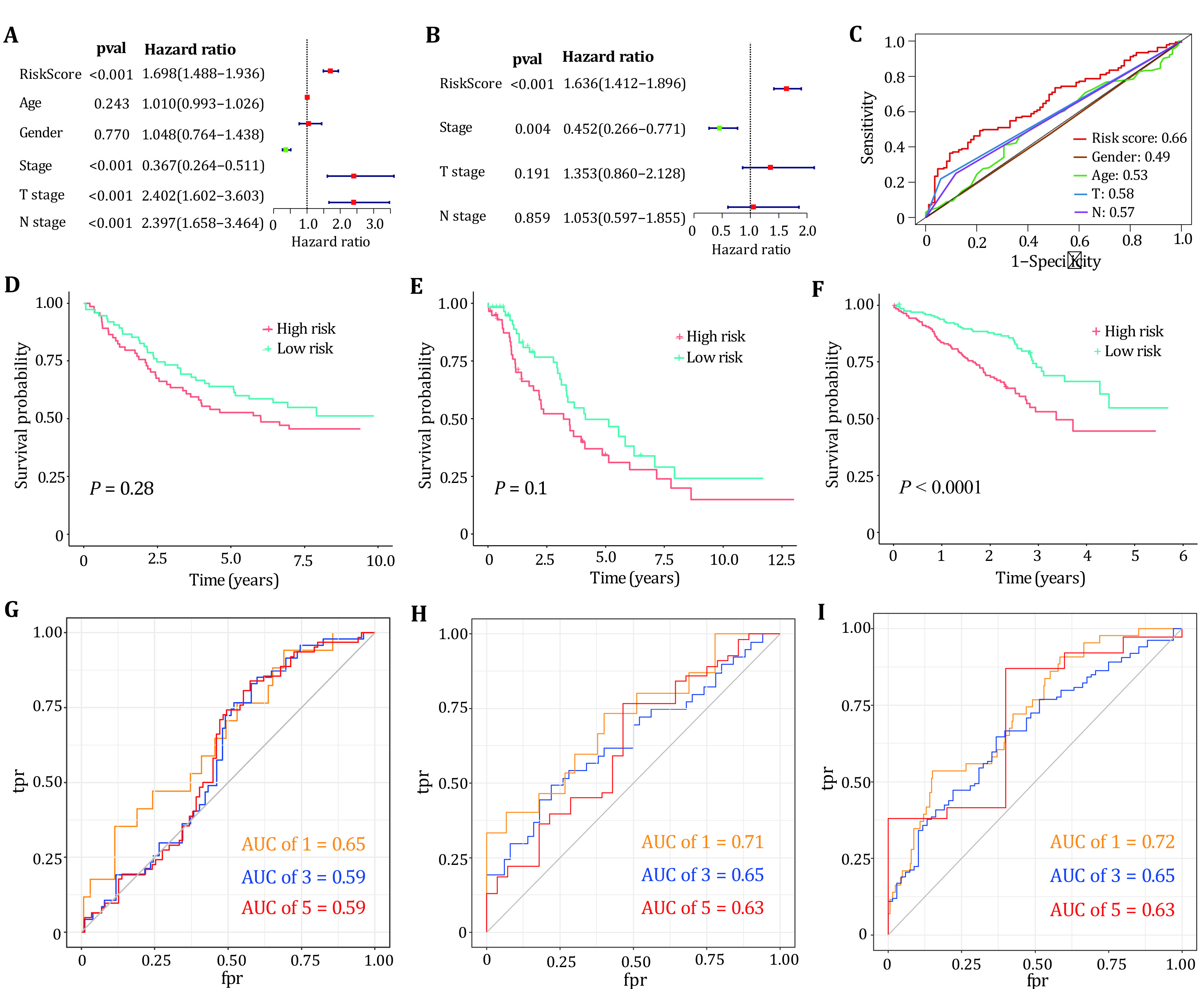
Validation of clinical features and models. **A**,**B** Univariate and multivariate Cox analyses of LUAD clinical parameters and prognostic models. **C** ROC curves for clinical characteristics and prognostic models. **D−F** Kaplan-Meier survival curves for three datasets GSE11969, GSE26939 and GSE72094. **G**−**I** The one-year, three-year and five-year ROC curves for the three data sets

In univariate and multivariate Cox regression analyses, the risk scores of five genes were significantly associated with poor prognosis in LUAD, suggesting that these factors are independent prognostic factors (Figs. 6A and 6B). Figure 6C plots the ROC curves of the risk score model, gender, age, T stage and N stage, respectively. The figure shows that our model has a better prediction effect relative to clinical characteristics. To further validate whether our prognostic model can be tested by independent validation and has cross-platform generality, three clinically informative microarray datasets GSE11969, GSE26939 and GSE72094 were obtained from the GEO database for external validation. Kaplan-Meier survival analysis displayed that our prognostic model can distinguish high-risk from low-risk groups in lung adenocarcinoma patients for the microarray dataset GSE72094 (Figs. 6D−6F). The one-year, three-year and five-year ROC curves of the three data sets are all above 0.5, which can better predict the survival rate of patients (Figs. 6G−6I).

## DISCUSSION

In our study, based on screened m6A-related immune genes, an immune gene prediction model for predicting survival in LUAD patients were constructed. The results indicated that the RNA methylation may play a key role in the progression of LUAD. Recent studies have shown that differential expression of m6A regulators plays a key role in the development of lung adenocarcinoma. The synergistic effect of multiple regulatory factors is a potential target for the treatment of lung adenocarcinoma. *FTO* and *METTL3* are key genes for the diagnosis and treatment of LUAD, among which the *METTL3* gene research is relatively detailed. The expression of *METTL3* was significantly up-regulated in lung adenocarcinoma, which promoted the malignant proliferation, migration and invasion of lung adenocarcinoma cells (Xu *et al.* 2022). *FTO* activates cell migration through m6A demethylation, thereby promoting the progression of LUAD cells (Ding *et al.* 2020). *YTHDC2* is down-expressed in lung adenocarcinoma, and is associated with the characteristics of poor differentiation, lymph node metastasis, tumor size and clinical stage of lung adenocarcinoma, and can inhibit the proliferation and migration ability of lung adenocarcinoma cells (Ma *et al.* 2021).

Studies have shown that immune cell infiltration and tumor microenvironment are related to tumor prognosis (Bai *et al.* 2022; Pagès *et al.* 2005). Key immune components associated with cancer patient survival have been described (Galon *et al.* 2006; Galon *et al.* 2007). These studies suggest that the introduction of immune-related parameters as prognostic factors may be clinically important. Mean immune-related gene expression by RNA-sequencing can reflect the status of immune cells and tumor cells and help to better predict patient survival. Many studies have shown that specific gene sets can predict the prognostic survival rate of cancer patients (Fatai and Gamieldien 2018; Guo *et al.* 2021), but the genes in the models are mostly immune-unrelated. With the development of tumor immunotherapy, the role of the immune system in cancer development is understudied, and how immune genes in tumor cells affect cancer prognosis is still poorly understood and poorly reported. We established and validated a prognostic model based on immune-related gene expression in tumor cells. Second, the five gene prediction models based on the immune gene dataset have cross-platform compatibility. We progressively reduced the number of differentially expressed genes in the prognostic model to five (*FCGRT*, *S100A16*, *SHC1*, *PAK4*, and *VIPR1*) by using the lasso algorithm and multivariate Cox regression analysis. Our immune prognostic model can classify lung adenocarcinoma patients into low-risk and high-risk groups, which can aid clinical decision-making by improving the accuracy of tumor staging and risk stratification. It has been widely reported that higher *S100A16* mRNA expression in lung adenocarcinoma was significantly associated with poorer survival, and the subcellular localization of *S100A16* and *S100A16* mRNA expression levels are prognostic markers in LUAD (Kobayashi *et al.* 2018). It had been reported that *SHC1* (SHC-adaptor protein 1) expression was closely associated with TMB, MMRs, MSI, TAMs, DNA methylation, m6A RNA methylation, tumor-associated immune infiltration and immune checkpoints in various cancers, and has been implicated in Carcinogenesis in cancer initiation, development and progression (Chen *et al.* 2022). It has been shown that p21-activated kinase 4 (*PAK4*), a serine/threonine protein kinase, was critical for cancer progression (Song *et al.* 2022). We propose an immune prognostic model based on RNA-seq of tumor samples, which provides an efficient assessment of the overall tumor immune status.

## MATERIALS AND METHODS

### Data processing

The RNA sequencing and mutation expression dataset as well as the corresponding clinical information of 594 lung adenocarcinoma patients were downloaded from TCGA (http://cancergenome.nih.gov/). The gene expression *FPKM* values of 535 lung adenocarcinoma (LUAD) samples and 59 normal samples were included. Then, the *FPKM* value was normalized to the *TPM* value by following the normalization formula for subsequent analysis.

1 $ {{TPM}}_{{i}}=\frac{{{FPKM}}_{{i}}}{\displaystyle\sum {{FPKM}}_{{j}}}\times {{10}}^{{6}}{(1}\leqslant  j \leqslant{56514)} , $

where the $ { {TPM}}_{ {i}} $represents the transcripts per kilobase of exon model per million mapped reads for gene *i*. $ { {FPKM}}_{ {i}} $ represents the fragments per kilobase of exon model per million mapped fragments for gene *i*. $ \sum { {FPKM}}_{ {j}} $ denotes the sum of the *FPKM* values of all genes.

In the validation phase, we screened lung adenocarcinoma microarray data from the Gene Expression Omnibus database (GEO; http://www.ncbi.nlm.nih.gov/geo/) with the search term "lung adenocarcinoma survival". Finally, three datasets containing clinical data are used for verification (GSE72094, GSE11969, and GSE26939).

### Selection and mutational analysis of 38 m6A regulators

By reviewing m6A related literature (Sun *et al.* 2019; Zaccara *et al.* 2019; Zhang *et al.* 2020a, 2021), we finally identified 38 m6A regulators, including 12 methyltransferases (*METTL3*, *METTL14*, *METTL16*, *WTAP*, *VIRMA*, *ZC3H13*, *RBM15*, *RBM15B*, *METTL5*, *CBLL1*, *ZCCHC4*, *PCIF1*), 23 binding proteins (*YTHDC1*, *YTHDC2*, *YTHDF1*, *YTHDF2*, *YTHDF3*, *HNRNPC*, *FMR1*, *LRPPRC*, *HNRNPA2B1*, *IGF2BP1*, *IGF2BP2*, *IGF2BP3*, *RBMX*, *EIF3A*, *ELAVL1*, *G3BP1*, *G3BP2*, *TRMT112*, *CPSF6*, *NXF1*, *SRSF3*, *XRN1*, *SETD2*) and three demethylases (*FTO*, *ALKBH5*, *ALKBH3*). First, we calculated the somatic mutation frequencies of 38 m6A regulators using the "maftools" R package. To determine whether genetic mutations affect the expression pattern of m6A regulators in LUAD samples, the mRNA differential expressions of 38 regulators between LUAD and normal lung adenocarcinoma samples were quantified and visualized as a heat map.

### Differentially expressed m6A-regulated genes

The m6A regulator expression information was extracted from 594 LUAD samples, and the differential expressions of m6A regulators between normal and tumor samples were calculated by the "limma" package. *P*-values were evaluated via the false discovery rate (FDR). The ratio of the average expression levels of genes in tumor samples to that in normal samples is defined as folding change. FDR < 0.05 and $ |{ {\log}}_2 {(folding\;\;change)|}  \geqslant   {0.25} $ were identified as the differentially expressed genes, which were thought to be associated with lung adenocarcinoma. Pearson correlation coefficients were used to assess the correlation between differentially expressed m6A regulators (Feng *et al.* 2019). Based on the differentially expressed m6A-regulated genes, the tumor samples were classified into different subtypes by using the Consensus Clust Plus software package. To make the subtype classification as accurate as possible, 50 iterations and a resampling rate of 80% were used. The number of clusters depends on the distribution of the cumulative distribution function (CDF). Since the results of PCA analysis did not clearly prove whether the classification is reasonable, the overall survival curves of different classifications were drawn, respectively, and the reasonable classification was determined by comparing the overall survival curves of different classifications.

### Analysis of immune cell infiltration and tumor immune microenvironment among different subtypes

First, the relative abundance of 23 immune cells in different subtypes was calculated using single-sample gene-set enrichment analysis (ssGSEA) (Subramanian *et al.* 2005). The ESTIMAIE algorithm was used to score stromal cells, immune cells and tumor purity in different subtypes (Yoshihara *et al.* 2013). The ESTIMATE algorithm includes two computing platforms: the "affymetrix" platform and the "illumina" platform. Here we use the "affymetrix" platform to analyze the patient's tumor microenvironment.

### Screening of m6A-related immune genes to establish a prognostic model

Based on the expression of genes in both subtypes A and B, differentially expressed genes between different subtypes in all tumor samples were found. The folding change is called the difference multiple. The 6364 genes with $  {|}{ {\log}}_{ {2}} {(folding\;\;change)|}  \geqslant   {2} $ and FDR < 0.01. The 1793 immune genes were downloaded from the ImmPort database. The Pearson correlation coefficients between different immune genes and different m6A regulators were calculated. The genes with coefficients greater than 0.1 and *P*.adj < 0.05 were called m6A-related immune genes. LASSO-COX regression analysis was used to screen for prognostic genes in m6A-related immune genes. The screened prognostic genes were further used to construct a risk score model. The formula for calculating the risk score is:

2 $ risk\;\;score = \sum\limits_{i \;= \;1}^n coe{f_i} \times  ex{p_i} . $

In the formula, *coef*_*i*_ represents the regression coefficient of the *i* gene, and *exp*_*i*_ represents the expression level of the *i* gene, where the regression coefficients are obtained from multivariate COX regression analysis. We calculated the risk score for each sample and divided lung adenocarcinoma samples into high-risk and low-risk groups based on the median risk score. The stability of the model is evaluated by external datasets.

## CONCLUSIONS

We demonstrated that the differential expression of m6A regulators in LUAD does not affect the mutational profile of m6A regulators. The tumor samples were divided into two subtypes and the detailed analysis of both subtypes was performed. We found a significant difference in survival between the two subtypes. By analyzing the tumor immune microenvironment in both subtypes, we found that the subtype with high immune infiltration had better survival with low tumor purity. We screened for m6A-associated immune genes and established a model that was associated with m6A and correlated with immunity. Finally, to improve the clinical application of the model, we analyzed clinical data through the model. These studies may contribute to the treatment and prevention of LUAD.

## Conflict of interest

Shujuan Li, Qianzhong Li, Luqiang Zhang, Yechen Qi and Hui Bai declare that they have no conflict of interest.
